# Tools for Sequence-Based miRNA Target Prediction: What to Choose?

**DOI:** 10.3390/ijms17121987

**Published:** 2016-12-09

**Authors:** Ángela L. Riffo-Campos, Ismael Riquelme, Priscilla Brebi-Mieville

**Affiliations:** 1 Molecular Pathology Laboratory, Department of Pathology, Faculty of Medicine, Universidad de La Frontera, Avenida Alemania 0458, 3rd Floor, Temuco 4810296, Chile; a.riffo.c@gmail.com (Á.L.R.-C.); ismael.riquelme@ufrontera.cl (I.R.); 2 Scientific and Technological Bioresource Nucleus (BIOREN), Universidad de La Frontera, Avenida Francisco Salazar 01145, Casilla 54-D, Temuco 4811230, Chile

**Keywords:** miRNA, bioinformatics, prediction tools, TargetScan, DIANA tools, miRanda

## Abstract

MicroRNAs (miRNAs) are defined as small non-coding RNAs ~22 nt in length. They regulate gene expression at a post-transcriptional level through complementary base pairing with the target mRNA, leading to mRNA degradation and therefore blocking translation. In the last decade, the dysfunction of miRNAs has been related to the development and progression of many diseases. Currently, researchers need a method to identify precisely the miRNA targets, prior to applying experimental approaches that allow a better functional characterization of miRNAs in biological processes and can thus predict their effects. Computational prediction tools provide a rapid method to identify putative miRNA targets. However, since a large number of tools for the prediction of miRNA:mRNA interactions have been developed, all with different algorithms, the biological researcher sometimes does not know which is the best choice for his study and many times does not understand the bioinformatic basis of these tools. This review describes the biological fundamentals of these prediction tools, characterizes the main sequence-based algorithms, and offers some insights into their uses by biologists.

## 1. Introduction

Non-coding RNAs are classified as long and small non-coding. The small non-coding RNAs in animals are composed of piRNA (24–30 nt in length), microRNA (~22 nt in length) and siRNA (~21 nt in length) [1]. The microRNAs (miRNA) are transcribed by RNA polymerase II from miRNA genes, generating a primary miRNA (pri-miRNA) that is then processed by the microprocessor complex to yield a precursor to miRNA (pre-miRNA) [2]. In some instances, pre-miRNAs are spliced out of introns from host genes and are then called mirtrons [3]. In a few cases, miRNAs are transcribed by RNA polymerase III [4]. Pre-miRNAs are exported to the cytoplasm and further processed by the DICER/transactivation response RNA-binding protein (TRBP) complex and finally by the RNA-induced silencing complex (RISC) [5,6]. The mature single-stranded miRNA acts as a post-transcriptional regulator binding to the mRNA in a complementary base-pairing manner to prevent the translation of this mRNA target [7].

miRNAs represent a novel epigenetic mechanism that regulates gene expression in many homoeostatic processes and pathological conditions within the cells. The dysfunction of miRNAs has been associated with a large number of diseases. For instance, the importance of miR-21 in different types of diabetes mellitus has been described by Sekar et al. [8], and the miRNAs of the hsa-let-7 family and others are associated with obesity and related metabolic diseases [9]. miRNAs also participate in arthritic diseases [10], kidney disease [11], cardiovascular diseases [12], etc. In the case of cancer, miRNAs are involved in all cancer types and can act as either tumor suppressors or inducers. Oncogenic miRNAs (oncomiRs) act directly on mRNAs from genes with pro-apoptotic or anti-proliferative roles. Conversely, tumor-suppressor miRNAs repress the expression of genes with oncogenic functions [13]. Therefore, RNA has been targeted for the study of new drugs and therapeutic methods [14,15,16].

However, the action of miRNAs on their mRNA targets is difficult to characterize, because each miRNA has multiple mRNA targets and vice versa; therefore, the correct identification of an interaction remains a challenge. The way to address this problem is usually through prediction and subsequent experimental validation of these miRNA:mRNA interactions. To this end, bioinformatic tools and new experimental techniques have emerged. Bioinformatic tools try to predict an effective miRNA:mRNA interaction for subsequent experimental validation. This knowledge enables progress to be made in elucidating the mechanisms by which the miRNAs act, both in normal physiological processes and in disease. In this regard, several tools for miRNA target prediction have been developed with different approaches for miRNA:mRNA interaction recognition. However, algorithms and parameters used by each tool are difficult to understand for scientists with little or no experience in the area of bioinformatics, making it difficult to choose the appropriate miRNAs for subsequent validation.

In this review we describe the principal biological fundamentals of the miRNA:mRNA interactions that the algorithms use for sequence-based prediction. We also describe the prediction methods used by the most frequently used tools TargetScan, miRanda and DIANA microT, explaining each parameter in the search results. Finally, we offer some considerations for using a miRNA analysis tool.

## 2. Biological Elements for Computational Analysis

**Figure 1:**
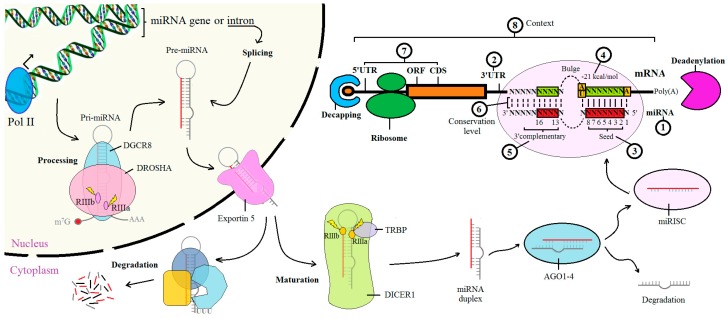
Biological basis used to predict how miRNAs interact with their mRNA targets by base pairing. After miRNA biogenesis, by DROSHA, Dicer and other protein regulators, the miRNA binds to its mRNA target and acts as a precursor to post-transcriptional gene silencing. The biological aspects used to predict these interactions are: (1) the miRNA sequence; (2) the 3′ UTR sequence; (3) the Watson–Crick base pairing in 5′ end of the miRNA, called seed; (4) the free energy expressed in kcal/mol; (5) the 3′ region of the miRNA that also have a Watson–Crick base pairing with the mRNA; (6) the level of conservation of this interaction between species; (7) other regions with Watson–Crick base pairing in the 5′ UTR, open reading frame (ORF) and coding sequences (CDS); and (8) other factors unrelated to the Watson–Crick base pairing that can affect the miRNA action, called context.

miRNAs regulate gene expression at post-transcriptional level through a complementary base pairing with the target mRNA, leading to mRNA degradation and translation blocking. These RNAs were discovered in the 1990s and three fundamental aspects of the miRNA functionality were promptly anticipated. First, miRNAs interact through a complementary antisense sequence with the mRNA targets [17] (Figure 1(1)). Second, the miRNA action area is delimited in the 3′ untranslated region (UTR) of the mRNA [17,18] (Figure 1(2)). Third, there are some conserved elements in the miRNA:mRNA interaction consisting of: a core of 9–7 nt in the 3′ UTR, which is complementary to the sequence core at the 5′ end of the miRNA (Figure 1(3)); the stacked G:C or G:U base in the RNA duplexes that form a bulge, which are associated with a decrease in the free energy (kcal/mol) in each RNA duplex (Figure 1(4)); a core site (5–9 nt) in the mRNA upstream, which is complementary to the 3′ of the miRNA (Figure 1(5)) [18].

The function of miRNAs has received special attention since 2001 [19] thanks to the publication of the initial results of the Human Genome Project [20,21], when new questions emerged about the number of genes and their definition. In the same year, responding to the need to decode the genomes, computational methods to predict structural components were developed, including tools to predict putative genes and their products, including the non-coding ones [22,23,24]. A large number of genes and their ncRNAs in different species were identified [25,26,27,28]. Some miRNA prediction software such as MiRscan estimated the number of miRNA genes between two and three hundred in the human genome, although not all were experimentally validated [29]. The number of identified miRNA genes increased rapidly and was expected to continue increasing, so new repositories were created to store sequences. In 2003, the Rfam database was created to store the RNA family sequences in various species identified and make this information available to the scientific community [30]. In addition, a uniform system was proposed for the identification and annotation of new miRNAs from diverse organisms [31]. In this regard, Rfam provides an online clearinghouse for miRNA gene name assignments.

The number of miRNA genes identified is continuously increasing, and many advances have been made in clarifying the mechanisms by which miRNAs act. In 2004, Lee et al. demonstrated that miRNA genes are transcribed by RNA polymerase II, which transcribes a long primary transcript either from miRNA genes or from introns of protein-coding genes (around 30%), producing a single miRNA or a small cluster of miRNAs containing two or more of these molecules [32]. Pri-miRNAs have a distinctive hairpin structure, capped in 5′ end and polyadenylated in 3′ end [32]. The DROSHA/DGCR8 (DiGeorge syndrome critical region 8) complex processes the pri-miRNAs to form the hairpin-shaped pre-miRNA. DROSHA contains two RNase III domains (RIIIa and RIIIb, see Figure 1), each of which cleaves to one strand of the pri-miRNA at the base of secondary structures, cutting off the single-stranded RNA in the 3′ and 5′ end to release ~60–70 nucleotide pre-miRNAs [33,34,35]. The pre-miRNA is then exported from the nucleus to the cytoplasm by Exportin 5 [36], where this pre-miRNA can be degraded or processed by DICER1, another RNase III enzyme. The two catalytic RNase III domains of DICER1 bind close to the terminal loop sequence of the pre-miRNA and perform the asymmetrical cleavage of the dsRNA stem, producing the mature ~22-nucleotide miRNA duplex. This process is assisted by TRBP, which also constitutes a physical bridge between the DICER1 complex and the Argonaute proteins (AGO1–4) to participate in the assembly of the miRNA-induced silencing complex (miRISC) [37]. The guide strand of the mature miRNA is bound by an Argonaute protein and retained in the miRISC to guide the complex to complementary target mRNAs for post-transcriptional gene silencing [7,38,39]. All this knowledge provides the biological basis to develop the bioinformatic tools needed for miRNA–target prediction. Each biological aspect is detailed below according to Figure 1.

### 2.1. The miRNA Sequences

All algorithms designed to search miRNA–target interactions require a miRNA sequence dataset. Thus, the more sequences added to this dataset, the more robust the tool is for miRNA-target prediction. The sequences from different species are deposited and/or obtained from databases such as Rfam that store non-coding sequences [40] and other miRNA-specific databases such as miRBase [41], TarBase [42] or miRecords [43]. In 2002–2003, when the first tools for miRNA-target prediction were created, there were only a few hundred miRNA sequences. For example, TargetScan used only 79 conserved mammalian miRNAs extracted from Rfam [44]. Back then, the programs estimated between 200 and 255 miRNA genes for the human genome [29]. The Encyclopedia of DNA Elements (ENCODE) project reported in 2012 that there were approximately 11,000 small RNA genes in the human genome [45], excluding those miRNAs encoded within introns. Currently, it is known that each miRNA is able to regulate from one to a large number of mRNAs and there are at least 1881 pre-miRNA and 2588 mature miRNA reported in the miRBase for humans (updated in June 2014), and a total of 35,828 mature miRNA products from 223 species stored in this database [41]. However, not all sequences reported in these databases have been experimentally validated and the exact number of miRNAs is far from being established. In this respect, there are a few curated databases, i.e., containing only information about validated miRNAs, as is the case with TarBase [46].

### 2.2. The 3′ UTR of mRNA

It is obvious that, apart from the miRNA sequences, the mRNA sequence is also necessary for the miRNA:mRNA prediction, especially in the 3′ UTR. Most programs use the 3′ UTR dataset to look for a target site, because many studies have shown that this area is the most frequently targetable in miRNAs. However, other experiments have shown that targeting can occur along the entire mRNA [47]. The sequence of a mRNA and its composition (e.g., start and end of 3′ UTR) can be obtained from the Ensembl database [48], the Reference mRNA Sequences (RefSeq) database [49] or the UCSC Genome database [50,51].

### 2.3. The Seed Region

The seed region comprises a zone between nucleotides 2 to 8, numbered from the 5′ to 3′ ends of a miRNA sequence, which has perfect Watson-Crick complementarity with the 3′ UTR of the mRNA. In 2003, Lewis et al. introduced this term as part of the search base of the TargetScan algorithm [52], and then the seed region was extended in the TargetScanS [52,53]. The seed region of a miRNA is a highly conserved segment that makes it possible to classify the miRNAs within families and species. For this reason, many tools such as PicTar [54], PITA [55], RNAhybrid [56], miRU [57] and others include the seed region as a key biological element for miRNA–target prediction. However, other algorithms use their own criteria for base pairing. This is the case with DIANA Tools, which uses miRNA-recognition elements (MREs), appealing to the fact that if the complementarity is partial, the stability of the target mRNA is not affected, but its translation is repressed (see below). Moreover, the existence of a G:U wobble in the seed region must be taken into consideration as this may affect the repression capacity of the miRNA [58].

### 2.4. Free Energy and Accessible Energy

Thermodynamic principles govern all reactions in biological systems. Therefore, the measurement of minimum free energy makes it possible to assess how strong the binding is between the miRNA and its target mRNA. Thus, the lower the free energy, the greater the RNA:RNA binding, increasing the likelihood that this interaction will actually occur [18,59]. The free energy is expressed in negative real value and its unit is kcal/mol. To measure this energy, the Vienna RNA package [60,61] is used for most miRNA:mRNA prediction tools. Moreover, programs such as PITA [55] use free energy to calculate accessibility to the binding site in 3′ UTR. This estimation is performed considering that the mRNA adopts a secondary structure, so the site to which a particular miRNA will join must be accessible [55].

### 2.5. miRNA 3′ Site

The 3′ site is an additional area with a Watson-Crick pairing at positions 13–16 nt at the 3′ end of the miRNA. This zone can contribute to the efficacy of gene downregulation. In addition, it can act as a compensatory interaction when there is a mismatch in the seed [53,58,62,63]. This parameter is included in TargetScan [53,63] and miRanda [64].

### 2.6. Conservation Status

When a binding site of a miRNA is maintained between different species, this site is deemed “conserved”. A conserved sequence can be present along any region within the miRNA sequence; the two most conserved areas tend to be the seed region and, to a lesser extent, the 3′ site of miRNA. The conservation analysis is performed using the phylogenetic and evolutionary distance calculations. This analysis aims to provide evidence that a predicted miRNA target is functional because it is being selected by positive natural selection. In this way, a higher degree of conservation arguably reflects a more reliable prediction [53]. The conservation level is an important parameter and is included in most algorithms for miRNA target prediction, including TargetScan [65], miRanda [64], DIANA-microT [66], PicTar [54], PITA [55] and EIMMo [67]. However, there are some exceptions, such as the RNA22 tool, which identifies putative target sites (target islands) independently of the conservation status [68].

### 2.7. Other Target Sites

Other mRNA sites in addition to the target site in 3′ UTR have been identified as targets for miRNA. These elements are the 5′ UTR [69,70], the open reading frames (ORFs) [71,72] and the coding sequence (CDS) for mRNAs [73], which have been included in the search of certain miRNA target prediction tools. For example, the new version of DIANA microT (v5.0) includes the prediction of miRNA:MRE recognition in the CDS region [66]. Moreover, the RNA22 tool includes the prediction of target islands in the 3′ UTR, 5′ UTR and CDS regions [68]. The miRTar [74] and TargetS tools [75] also include these three regions in their predictions.

### 2.8. The Contexts

This parameter refers to those factors that may affect the miRNA:mRNA interaction, but that do not directly involve a sequence-based recognition pattern. Among these factors some regulatory processes, such as the binding of proteins to RNA [76] and the methylation of several RNA sites [77]. The differential targeting of miRNAs among different cell lines or tissues [65] are also included as context parameters. Finally, the presence of long non-coding RNAs (lncRNAs), which can act as inhibitory decoys of miRNAs through miRNA:lncRNA interactions [78,79] are also regarded within the context. Some tools for miRNA target-site prediction that have a sequence-based recognition system come with the context parameters incorporated. For instance, parameters such as the degree of repression and the cooperative miRNA function are included in the TargetScan algorithm [63,65,80]. In addition, the alternative splicing parameter is considered by the miRTar tool [74], and the secondary structure accessibility is considered by the miRanda tool [81] and PITA [55]. By contrast, there are advanced tools designed to infer the expression-based miRNA targets [82,83], or the network-based methods to detect miRNA regulatory modules [84,85], but these are not discussed in this review.

## 3. Bioinformatic Tools for miRNA Target Prediction

On the biological basis described above, in the last 15 years, many bioinformatic tools have been developed to predict miRNA:mRNA interaction. These tools were developed to identify quickly and accurately those target miRNAs with a potential cellular role to enable their functional characterization and validation in a biological model. In general, bioinformatic tools can be grouped according to the platform used in web-based services, downloaded software and packages (in this case for R, see Table 1). The web-based service is the most user-friendly platform, because it usually allows for a simple data entry with few options for modification and concrete output information. Downloaded programs are the next level in the order of complexity because they also have a user-friendly environment, but they allow users more treatment options for processing data. Finally, the most complex platforms are the packages, because they are not designed to be user-friendly and require knowledge in bioinformatics. However, packages offer advantages such as greater freedom, analysis improvement and adaptation, versatility in programming language (e.g., Python or Java), and they can be obtained from repositories such as GitHub (Available online: https://github.com/), the Comprehensive R Archive Network (Cran, available online: https://cran.r-project.org/), and others. As the web-based tools are the most frequently used platform to predict miRNA:mRNA interaction based on sequence, in this review three of the most complete and widely used sequence-based tools and their algorithms will be described next. For more features in the other tools, see [86,87,88].

### 3.1. TargetScan

In 2003, Bartel′s group developed TargetScan, becoming the first algorithm used to predict miRNA targets in vertebrates [44]. Since then, this tool has been improved through new versions and additional algorithms that have helped to improve match prediction accuracy: TargetScanS [52], TargetScan P_CT_ [53], TargetScan context+ score [63,65,80]. All of these are described below. These web-based tools have two search options: the gene symbol and/or the species-specific miRNA name (human, rhesus, mouse, rat, cow, dog, opossum, chicken, frog, worm, fly and fish). In the search by gene symbol, the results are shown by its different transcripts—if this gene has more than one—and are classified as more or less prevalent. For each transcript, the sites with higher and lower probability of targeting by miRNAs are displayed. This probability is estimated by including all the algorithms and parameters (site type, context++ score, context++ score percentile, weighted context++ score, conserved branch length, and P_CT_) for each miRNA candidate. However, when searching for the name of a miRNA, the results show the target genes according to the specific transcript. In this case, the probability can be ranked by: aggregate P_CT_, cumulative weighted context++ score or only with conserved sites. To understand the results it is necessary to understand each one of these parameters (see Table 2).

The first version of TargetScan [44] used the Rfam [30] database to get the miRNA sequences. These sequences were contrasted with other genomes, creating a miRNA dataset from mammalian (79 miRNA seed families) and vertebrate genes (55 miRNA seed families). Parallel to the above-mentioned study, an 3′ UTR dataset for mammalian and vertebrate genes was created to identify miRNA:target interactions. These interactions are exerted in segments of 7 nt with perfect Watson-Crick complementarity to bases 2–8 of the miRNA, numbered from the 5′ end. The area with perfect Watson-Crick complementarity was called the seed matches. For this, TargetScan was considered: the expected frequency of seed matches in the 3′ UTR dataset; the expected frequency of matching to the 3′ end of the miRNA; the observed count of seed matches in the 3′ UTR dataset, optimized base pairing of the remaining 3′ portion of the miRNA to the 35 bases of the UTR immediately 5′ from the seed match; the predicted free energy of a seed:seed match duplex (kcal/mol), assigning a Z score to each 3′ UTR. False positives were corrected and process controls were used at every step. After the analysis, 451 mammalian miRNA:target interactions (from 400 genes) and 115 vertebrate miRNA:target interactions (from 107 genes) were identified. For each miRNA an average of 3.9 targets was predicted [44].

**Figure 2:**
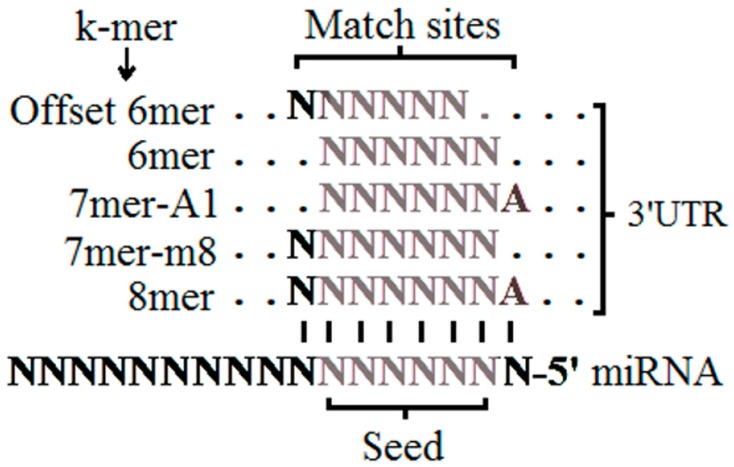
Sites matching in the miRNA seed region, including all k-mer: 8mer, 7mer-m8, 7mer-A1, 6mer, and offset 6mer (Figure adapted from [46]).

Lewis et al. developed TargetScanS in 2005 [52], including new genomes (chicken and dog), and thus reduced the number of false positives. The seed region was extended—allowing for the sequence that flanks this seed region—to four site types, increasing the specificity of the miRNA:target interaction. These four match sites in the seed region include one 6mer, two 7mers, and one 8mer, where k-mers refer to all the possible subsequences of length k, from a read or sequence. The 6mer is the perfect 6-nt match to the miRNA seed, from the position 7 to 2 nt. The 8mer site comprises the seed match from 8 to 2 nt and include the A at position 1. The 7mer-m8 site comprises the seed match from the 8 to 2 nt. The 7mer-A1 is the seed match comprising from 7 to 2 nt and includes the A at position 1 (see Figure 2). In this way, varying the match seed used also changes the number of targets identified per miRNA. The k-mers can be sorted according to site efficacy in 8mer > 7mer > 7mer-A1 > 6mer [52].

The seed matches are not always sufficient to confer repression and if the repression occurs, the grade of repression is highly variable in different UTR contexts. To solve these issues, Grimson et al. created in 2007 the TargetScan context+ score algorithm, which incorporated five general features of site context to enhance the site efficacy [63]. The first of these site contexts is represented by the number of A and U nucleotides that may be immediately flanking the seed and that are strongly associated with the functional site and influence site efficacy. The local AU context can be associated with a weaker mRNA secondary structure near the site and thus an improvement in the accessibility to the seed site. The second site context involves the position where the effective sites are located. For example, no detectable efficacy in 5′ UTRs, detectable but marginal efficacy in ORFs, and high efficacy in 3′ UTRs were observed. Therefore, the effective sites preferentially reside in the 3′ UTR, but not in the segment immediately following the stop codon. The third context involves the effective sites that preferentially reside near both ends of the 3′ UTR. The UTR quartiles near the ORF and near the poly(A) tail were more susceptible to effective targeting. The fourth context is the additional Watson-Crick pairing site at nucleotides 12–17 in the 3′ portion of the miRNA. When pairing occurs in the 3′ portion, more specifically at 13–16 nt of the miRNA, an enhanced repression of canonical 7- or 8mer sites can happen, decreasing the dissociation rate of the bound silencing complex. Finally, the last site context is represented by the proximity of other binding sites within an mRNA sequence for other co-expressed miRNAs, which leads to cooperative action. This cooperative miRNA function involves a mechanism through which repression can become more sensitive to small changes in miRNA levels. The effects of each context feature could be considered independently. In summary, the different contexts provide valuable information for selecting which of the many mammalian miRNA:target relationships are the most promising for experimental follow-up [63].

In some cases the perfect seed-pairing is not a reliable predictor of miRNA:target interactions. This is the case with the AU-rich seed regions, which can decrease the stability of seed-pairing interactions. Additionally, the AU-rich seed regions have more 3′ UTR binding sites, which could dilute the effect on each target message. Therefore, in 2011 Garcia et al. expanded the TargetScan tool to predict miRNA regulation quantitatively so as to model differential miRNA proficiencies, thereby improving prediction performance. They incorporated predicted seed-pairing stability (SPS) and target-site abundance (TA), concluding that both parameters have a substantial impact on targeting proficiency and can be used to improve miRNA:mRNA prediction. Moreover, the authors incorporated the multiple linear regression models for context-only and computed context+ using the lm() function in the R package [80].

In 2009 Friedman et al. developed an algorithm of preferentially conserved targeting (P_CT_) called TargetScan P_CT_, which is an improved method for quantitatively evaluating site conservation (TargetScan v5.0) [53]. This new algorithm incorporates a phylogenetic tree based on the UTR genomic regions, using a branch-length metric to evaluate motif conservation. This tool gathers the genomes of 28 vertebrate species and a new match site in the seed: the offset 6mer site (see Figure 2). Furthermore, substantial improvements were introduced to the estimation of the conservation-specific backgrounds of individual seed-match sites. This makes statistically sound comparisons possible between the conservation of seed-match types, between seed matches to different miRNAs, and even between individual sites. They also studied the possibility of selective conservation of imperfect seed matches, concluding that mismatched seed sites are hard to be selectively maintained and that these compromised the prediction specificity. On the other hand, the pairing within the 3′ portion of the miRNA was also studied, concluding that this site in 3′ can compensate for a single-nucleotide bulge or mismatch in the seed region. For this reason, these sites were called 3′ compensatory sites. The P_CT_ is calculated for each of the five seed-match sites and the values range between 0 and 1. In addition, the P_CT_ correlates with the mean level of mRNA destabilization and provides a useful criterion for assessing the biological relevance of predicted miRNA–target interactions (the higher the site conserved, the greater the destabilization). The P_CT_ values and the context scores provide independent and complementary information, useful for predicting the biological relevance and efficacy of each site [53].

In 2014, Nam et al. studied the repressed genes and their isoforms for the same miRNAs in different cell lines and tissues [65]. Hence, the term affected isoform ratio (AIR), which indicates the fraction of mRNA transcripts containing a target site for each miRNA, was incorporated. They also developed a revised prediction model, called weighted context+ or wContext+, which was also included in TargetScan. This model yields a cell-type-specific score for each site, calculating first the score for each of the context [80] and then weighting the score by the AIR of a specific site in each cell type. The scores from multiple sites are added to the yield of the total wContext+ score. The scores with lower negative values indicate greater predicted repression [65].

In the latest version of TargetScan (v7.0), Agarwal et al. suggest that only the canonical sites of the seed region have an effect on the drop in mRNA levels and the non-canonical sites have no effect on mRNA degradation [89]. Based on this, 14 features (3′ UTR target site abundance, predicted seed pairing stability, identity of nucleotide at position 1 of the sRNA, identity of nucleotide at position 8 of the sRNA, identity of nucleotide at position 8 of the site, local AU content near the site, 3′ supplementary pairing, predicted structural accessibility, minimum distance of site from stop codon or polyadenylation site, probability of conserved targeting, ORF length, 3′ UTR length, number of offset-6mer sites in the 3′ UTR and number of 8mer sites in the ORF) were used. In order to relate the 14 features, multiple linear regression models were applied to each of the four site types (8mer, 7mer-m8, 7mer-A1, and 6mer) and collectively called the “context++” model of miRNA targeting efficacy [89].

### 3.2. miRanda

The miRanda algorithm was developed by Enright et al. in 2003 and designed to find potential target sites for miRNAs in the genomic sequence [81]. The miRanda algorithm was included in a miRNA web-based tool [90]. This tool permits searches by the name of the miRNA and the symbol for the mRNA in a selected organism (*Homo sapiens*, *Mus musculus*, *Rattus norvegicus*, *Drosophila melanogaster* and *Caenorhabditis elegans*), visualizing all miRNA expression levels for a given set of tissues in humans, mice and rats. Moreover, this website allows both the miRNA dataset and the miRNA target prediction software to be downloaded with an open-source license, and researchers can adjust the algorithm. As a result of a target mRNA search, the complete gene sequence, the target sites of each miRNA and the miRNA:mRNA interactions are displayed as sorted by the mirSVR score and PhastCons score (see Table 2) [64,91]. When a specific miRNA is searched, however, a list of the corresponding potential target mRNAs is obtained, ordered according to the mirSVR score. miRanda also indicates the alternative isoforms when appropriate [91].

The initial miRanda algorithm was based on the local alignments of miRNA:UTR, assessing the thermodynamic folding energy of a miRNA:UTR duplex [81]. The local alignment is an adaptation of the Smith-Waterman algorithm [92] based on the complementarity of nucleotides (A=U, G≡C or G=U) between miRNA (*D. melanogaster* dataset from Rfam) and a 3′ UTR (dataset from Berkeley Drosophila Genome Project). To this end, miRanda uses a scoring matrix for the individual alignment, assigning values and penalties for each base complementarity: +5 for G≡C, +5 for A=U, +2 for G=U and −3 for all other nucleotide pairs, −8 for gap-opening and −2 for gap-extension. The known target sites at the first eleven positions are multiplied by a scaling factor (here set at 2.0) so as to reflect the observed 5′–3′ asymmetry. These and other considerations are used to obtain the complementarity score between the miRNA and mRNA sequences (typically at 3′ UTR). Additionally, the authors use the Vienna package to calculate the thermodynamic folding energy (kcal/mol) of optimal strand-strand interaction between miRNA and UTR [61]. The total scores for each interaction is corrected by the criterion of the evolutionary conservation of target sites in fly [81].

Moreover, this miRanda algorithm was included in the miRNA web-based tool and optimized for use with human, mouse and rat data [90]. The miRanda tool can be also downloaded with an open-source license and researchers can adjust the algorithm. To estimate the probability that a predicted site is incorrect, a shuffled miRNA was obtained by randomly swapping (1000 times) selected base pairs with a constant nucleotide composition. Subsequently, these shuffled miRNAs were scanned against human, mouse and rat 3′ UTR sequences. The false-positive rate was obtained by comparing the scores of shuffled miRNAs with real miRNAs. The authors predicted that close to 9% of all mammalian genes have more than one miRNA target site in their 3′ UTRs and 1314 gene candidates with more than two target sites [90].

The authors proposed that the seed region is not the only parameter that must be used for the analysis of miRNA target, as there is a large number of non-canonical sites, i.e., those sites without perfect seed complementarity. Moreover, some studies have shown that changes in mRNA expression are reasonable indicators for miRNA regulation. On this basis, in 2010 Bethel et al. incorporated the miRNA support vector regression algorithm (mirSVR) for scoring and ranking the efficiency of miRanda-predicted miRNA target sites, considering the mRNA expression changes [64]. mirSVR incorporates target site information and contextual features derived from the miRanda-predicted miRNA:site duplex for the local and global context of the 3′ UTR sites without the need to define seed subclasses (Figure 2). Local features include the AU composition flanking the target site and the secondary structure accessibility score. Global features include length of UTR, relative position of target site from UTR ends, and conservation level of the block containing the target site (by phastCons scores) [64]. The phastCons scores measure the conservation of nucleotide positions across multiple vertebrates [64,93]. Thus, the miRanda tool detects genes effectively with either non-conserved or conserved sites.

### 3.3. DIANA Tools

DIANA Tools is a web service that provides access to the tools and data resources for miRNA analysis. The tools for miRNA target prediction use the microT algorithm [94] and subsequent improvements. Currently, the microT-CDS algorithm (v5.0) and the microT v4 algorithm are available. The latter version supports two new species, *Drosophila melanogaster* and *Caenorhabditis elegans.* A gene or a miRNA can be specified in the search field and in all cases a KEGG description can be included and the species can be chosen. This procedure on the web is the same for both the microT-CDS and microT v4 algorithms. However, as expected, the results are presented differently for each algorithm. In microT-CDS many results are shown in a different order of importance compared to microT v4, and parameters such as SNR (signal-to-noise ratio) and the precision score have been removed, being sorted only for the miTG score (miRNA targeted genes) (see Table 2). The detailed information includes the analyzed region, binding type (k-mer), position of the miRNA interaction in the transcript, specific score, sequence of binding area, the numbers of conserved species, and the chromosome position.

The DIANA-microT tool for human and mouse miRNA target prediction was developed in 2004 by Hatzigeorgiou′s group [94]. The microT algorithm uses miRNA-recognition elements (MREs) for the RNA:RNA base pairing (miRNA:MRE recognition). The MREs are searched in the 3′ UTRs of human mRNAs extracted from the RefSeq database [49,95]. The authors included two parameters in the search algorithm, the first being the calculation of the free energy of the canonical Watson-Crick pairing and G-U wobble dinucleotide base pairs for the putative MRE identification. For this reason, a window of 38 nt that slides over the mRNA sequence was used to calculate the minimum binding energy between the miRNAs and sequences for every three consecutive nucleotide pairs in the human 3′ UTR database. The second parameter involves miRNA-associated protein(s) (miRNP) that impose restraints on the position and sizes of the loops and nucleotide bulges between miRNAs and their cognate MREs.

Subsequently, in the DIANA-microT v3.0 miRNA target prediction algorithm, the miTG score, SNR, precision score and KEGG description were included [96]. The MREs can be UTR sites of 7, 8 or 9 nt in length with a consecutive WC base pairing with the miRNA (7mer, 8mer, and 9mer, respectively), starting from position 1 or 2 (from the 5′ end). The MREs also incorporate sites with an additional base pairing at the 3′ end of the miRNA and a single G:U wobble pair (miRNA bugle) or binding of only six consecutive nucleotides (6mer) to the driver sequence (see Table 2). The features the MRE binding type and conservation profile were measured using up to 27 species to evaluate each miRNA or each predicted MRE interaction. This software compares these measurements with those predicted for a set of mock miRNAs. This evaluation makes it possible to calculate a miRNA-specific SNR at different miTG score cut-offs, which enables identification of the current interactions without background noise. The algorithm can also estimate a precision score that serves as an indicator of the false-positive rate in a particular miTG interaction. The overall miTG score is calculated as the weighted sum of the scores of all identified MREs on the 3′ UTR [96].

Later, in the microT-v4 algorithm, the authors included two additional species (*D. melanogaster* and *C. elegans*) and updated the information extracted from miRBase (v13) and Ensembl (v54) for human, mouse, fly and worm species. This algorithm also includes a bibliographic analysis that correlates miRNAs with different diseases, a graphic display with all the relevant functional information from the UCSC genome browser and a tracker for changes in the miRNA nomenclature [97].

In the latest version of microT (web server v5.0) the algorithm was improved (DIANA-microT-CDS) [66] and the web server was completely redesigned [98]. Considering the studies showing that a protein-coding sequence of an mRNA can be a target with a miRNA with a measurable effect on miRNA-mediated mRNA degradation, the microT-CDS algorithm includes the analysis of MREs in this region. MREs are defined from sequencing data and the conservation score of a MRE is based on the alignments of 16 vertebrate genomes. In addition, a dynamic programming algorithm identifies the optimal alignment between the miRNA-extended seed sequence (1–9 nt from the 5′ end of miRNA) and every 9 nt window on the 3′ UTR or CDS. A separate prediction model is built for the 3′ UTR and CDS regions, and then these are combined to calculate the final miRNA:mRNA interaction score [66]. These and other changes (see [98]) have been incorporated into the web server and the database has been updated in miRBase v18 and Ensembl v69.

## 4. Discussion

### Considerations for Using a miRNA Analysis Tool

The tools to predict miRNA:mRNA interactions are designed to obtain accurate results and decrease the false-positive rates. Nevertheless, the effective prediction of this interaction remains challenging. The limited knowledge of the rules that govern the mechanism of mRNA degradation by miRNA does not facilitate validation of the total predicted interactions. In this regard, it is essential to weigh the biological aspects used by each prediction tool. Each program has its advantages and weaknesses, and the choice depends on the individual researcher′s requirements.

In general, it can be said that some prediction tools prioritize the elimination of false positives from the list of candidates, i.e., they seek more “recision” in the prediction, but they have the disadvantage of omitting from the results miRNAs that may be involved (false negatives). While other programs are far too permissive, i.e., they endeavor to increase the “sensitivity”, they have the disadvantage of including a large number of false positives.

In this sense, TargetScan is the most precise of the sequence-based tools, but with a high false negative rate that leads to low sensitivity. By contrast, miRanda has greater sensitivity, but a higher false positive rate. Instead, the DIANA-microT attempts to be balanced and also includes a parameter to compare its predictions to those from other tools. This comparison is reflected in the number of predicted interactions for each program according to a single miRNA or mRNA, e.g., for a determined miRNA, miRanda can predict a greater interactions number (7982) than either TargetScan (1367) or DIANA-microT (961) predictions.

When researchers want to know which miRNAs may be involved in the regulation of the human *KRAS* gene for example, TargetScan is at a significant advantage because this tool incorporates more complete information about the number of isoforms than miRanda, and DIANA-microT does not differentiate between isoforms. Thus, TargetScan should be used to know which miRNAs are interacting with a particular isoform. The search results for the *KRAS* gene indicate that the predicted interactions do not match in the three tools. In the case of miRanda, the most likely interaction is between miR-181d and the 4626–4650 position in the canonical *KRAS* isoform, with a mirSVR score of −1.1718. In TargetScan, the most likely interaction is between the miR-183 and the 4328–4335 position in the canonical *KRAS* isoform, with a context++ score of −0.57 and a P_CT_ score of >0.99. In DIANA-microT, the most likely interaction is between miR-32 and the 4938–4953 position in the mRNA of *KRAS*, with a miTG score of 0.999. DIANA-microT also verifies whether the predicted interaction has been validated experimentally and whether TargetScan can also predict it. However, TargetScan predicts this interaction as a poorly conserved interaction outside the seed region. Accordingly, TargetScan′s context++ score is low (−0.21) and located at another position (183–190) of the mRNA.

A lower percentage of interactions is predicted by more than one bioinformatic tools and these interactions are not usually located at the same molecular position. For example, miRanda and TargetScan could predict that miR-181d potentially bind *KRAS* mRNA, but at different positions of the mRNA sequence. This phenomenon may occur because these tools use different miRNA and mRNA databases and versions that cause discrepancies in the predictions. The latest version of DIANA-microT uses miRBase v18 and Ensembl v69, whereas miRanda uses the 2010 versions of Rfam and Ensembl, and TargetScan uses miRBase v21 and RefSeq/Gencode. The database differences can cause variations in the number and types of miRNA or mRNA, in nomenclature and in the origin of the sequence (predicted or curated). In this respect, the latest version of TargetScan uses the most current databases, followed by DIANA-microT and miRanda tools, respectively.

The biological aspects are also important in the prediction algorithms (see previous section for detailed description). For instance, TargetScan is stricter about the interaction site, since it considers only the seed region and the 3′ UTR in the search and does not support mismatches. In addition, it heavily prioritizes the conservation level of miRNA:mRNA interactions and rejects the interactions in ORFs and 5′ UTR regions as ineffective at inducing repression. This consideration is reflected in the P_CT_ and context++ scores, complementary to one another, that make it possible to prioritize the choice of predicted interactions. Typically, the same P_CT_ score is obtained for multiple miRNA:mRNA interactions, in which case researchers should consider the context++ score to prioritize the choice of interactions. Conversely, when the same context++ score is obtained for multiple miRNA:mRNA interactions, the P_CT_ score should be taken into consideration for the purposes of prioritization. Moreover, the cell type also helps in the selection of the most likely interaction, since it has been seen that expression of the RNAs is linked to the cellular context. In this regard, both DIANA-microT and TargetScan make adjustments in their scores for engaging this context. TargetScan is not appropriate when trying to obtain new interaction sites or sites that do not have a strong selective pressure. RNA22 tool is an option for searching for new miRNA:mRNA interactions, because its predictions are independent of the state of conservation and also includes interactions along the entire mRNA (3′ UTR, 5′ UTR and CDS regions), but at the risk of a large number of potential false positives. The miRanda and DIANA-microT tools could also be helpful because they analyze non-conserved sites and MREs, which can also comprise CDS interaction sites. It is also important to note that the existence of a miRNA:mRNA interaction does not guarantee the biological repression of a specific mRNA. Thus, DIANA-microT includes a correction in its score that analyzes the effect on the miRNA-mediated degradation of mRNA. The miRanda score considers the calculation of the energy needed to access the binding site, while TargetScan considers the context.

In summary, which is the most reliable tool for making the best prediction? Of the three tools described, TargetScan seems to be the most robust tool, because it enables a more complete search at isoform level, it penalizes the less conserved interactions, and its databases are the most up-to-date. For these reasons, TargetScan can predict miRNA:mRNA interactions with a higher probability of being biologically validated than the other tools. However, the algorithms used by miRanda (mirSVR score) and DIANA-microT (miTG score) can complement these prediction studies, as they take additional biological parameters into consideration that remain interesting to verify.

Moreover, in order to increase the choice of candidates with the greatest likelihood of being experimentally validated, the use of more than one tool is recommended. For this reason, DIANA-microT reaffirms its prediction, indicating that other tools can also predict the same interactions. For instance, there are tools that allow these comparisons, such as miTAE ([99,100], available online: http://cbl-gorilla.cs.technion.ac.il/miTEA/) and mirDIP ([101], available online: http://ophid.utoronto.ca/mirDIP/), but this strategy is not enough. In recent years, thanks to the large amount of data from massive sequencing technologies, new tools have been created for miRNA:target prediction. These new tools attempt to explain statistically the observed expression patterns and include information about relationship networks in order to detect the miRNA regulatory module, thereby improving the prediction accuracy (for more information see [102]). Moreover, the use of scripts and packages provides more precise miRNA target prediction for each particular case. Despite all this information, it is important to bear in mind that of all the miRNA genes predicted currently, only ~25% have been validated [102], meaning there is no guarantee that all the predictions made by these tools can be validated biologically.

## 5. Conclusions

In this review, the biological bases used to predict miRNA:mRNA interactions were explained (Figure 1) in order to gain an overall view of the importance of these elements as part of the prediction. Three of the most used web-based tools were described here: TargetScan, miRanda and DIANA-microT. This detailed explanation contributes to understanding how each tool arrives at its score (Table 2), aiding in interpreting the results and in understanding how each tool prioritizes its predicted interactions. Finally, the advantages and disadvantages of these tools were described, highlighting TargetScan as the best option in most cases. However, miRanda and DIANA-microT can be used as complementary algorithms, since they consider additional parameters that TargetScan does not prioritize, and they might be appropriate in cases of non-conserved interaction, rare sites or other situations where repression can also occur. The need to move towards the use of new tools to predict miRNA:mRNA interactions, e.g., network-based prediction tools, requires a deeper exploration.

## Figures and Tables

**Table 1:** The most relevant web-based tools for miRNA sequence-based prediction. Some programs and R-packages for miRNA analysis.

Type	Name	URL
Web-based	TargetScan	http://www.targetscan.org/
	Diana Tools	http://diana.imis.athena-innovation.gr/DianaTools/index.php
	miRanda	http://www.microrna.org/microrna/getGeneForm.do
	PITA	http://genie.weizmann.ac.il/pubs/mir07/mir07_prediction.html
	PicTar	http://pictar.mdc-berlin.de/
	RNA22	https://cm.jefferson.edu/rna22/
	RNAhybrid	http://bibiserv.techfak.uni-bielefeld.de/rnahybrid/
	miRTar	mirtar.mbc.nctu.edu.tw/
	TargetS	http://liubioinfolab.org/targetS/mirna.html
	miRU	http://plantgrn.noble.org/psRNATarget/
	EIMMo	http://www.mirz.unibas.ch/
Downloadable programs	miRPlant	http://sourceforge.net/projects/mirplant/
	MiRNA-EMBL	http://www.russelllab.org/miRNAs/
	miRspring	http://mirspring.victorchang.edu.au/
	miRNA Digger	http://www.bioinfolab.cn/
	miRanda	http://www.microrna.org/microrna/getGeneForm.do
	miRge	http://atlas.pathology.jhu.edu/baras/miRge.html
R-packages	microRNA	https://bioconductor.org/packages/release/bioc/html/microRNA.html
	miRNApath	https://bioconductor.org/packages/release/bioc/html/miRNApath.html
	AgiMicroRna	https://bioconductor.org/packages/release/bioc/html/AgiMicroRna.html
	mirIntegrator	https://bioconductor.org/packages/release/bioc/html/mirIntegrator.html
	miRNAtap	https://bioconductor.org/packages/release/bioc/html/miRNAtap.html
	TargetScore	https://bioconductor.org/packages/release/bioc/html/TargetScore.html
	ExiMiR	https://bioconductor.org/packages/release/bioc/html/ExiMiR.html
	LVSmiRNA	https://bioconductor.org/packages/release/bioc/html/LVSmiRNA.html
	MiRaGE	https://bioconductor.org/packages/release/bioc/html/MiRaGE.html
	miRcomp	https://bioconductor.org/packages/release/bioc/html/miRcomp.html
	miRLAB	https://bioconductor.org/packages/release/bioc/html/miRLAB.html
	miRNApath	https://bioconductor.org/packages/release/bioc/html/miRNApath.html
	miRNAtap	https://bioconductor.org/packages/release/bioc/html/miRNAtap.html
	MmPalateMiRNA	https://bioconductor.org/packages/release/bioc/html/MmPalateMiRNA.html
	oneChannelGUI	https://bioconductor.org/packages/release/bioc/html/oneChannelGUI.html
	RmiR	https://bioconductor.org/packages/release/bioc/html/RmiR.html
	Roleswitch	https://bioconductor.org/packages/release/bioc/html/Roleswitch.html
	ssviz	https://bioconductor.org/packages/release/bioc/html/ssviz.html

**Table 2:** Description of the parameters and values shown in the results delivered by TargetScan, miRanda and DIANA-microT tools.

Tools	Parameter	Value Range	Meaning
TargetScan	Site type	8mer > 7mer > 7mer-A1 > 6mer	The matching sites in the seed region (nucleotides 2 to 8 from 5′ of miRNA that have perfect WC pairing with the 3′ UTR), from the strictest to the least strict.
	Context++ score	From 1 to −1	The sum of the contribution of 14 features for each of the four site types, the more negative the score, the greater the repression.
	Context++ score percentile	From i to 100 − i;	Percentage of sites for the miRNA with a less favorable context++score.
	Weighted context++ score	From 1 to −1	The scores with a lower negative value indicate a greater prediction of repression.
	Cumulative weighted context++ score	C(i–1) + (1 − 2CSi)(AIRi-C(i–1))	This score estimates the total repression expected from multiple sites of the same miRNA, for each mRNA target predicted.
	Branch-length score	8mer: 1.8; 7mer-m8: 2.8; 7mer-A1: 3.6; 6mer: NA	This score is the sum of phylogenetic branch lengths between species that contain a matching site.
	P_CT_ score	Between 0 and 1	The higher the score, the greater the conservation and the greater mRNA destabilization expected.
	Aggregate P_CT_	Value = 1 − ((1 – P_CT_) site1 × (1 − P_CT_) site2	For each miRNA, this parameter includes the conserved 3′ UTR targets with multiple sites that were missed in the human 3′ UTR annotation, but were present in the mouse annotations.
	Conserved sites	≥0	Number of conserved sites identified.
miRanda	mirSVR score	<0	This score is an estimate of the miRNA effect on the mRNA expression level. The more negative the score, the greater effect.
	PhastCons score	From 0 to 1	This measures the conservation of nucleotide positions across multiple vertebrates.
DIANA Tools	miTG score	From 0 to 1	This is a general score for the predicted interaction, the closer to 1, the greater the confidence.
	Also Predicted	red, blue and green	This compares with other tools; miRanda in red, TargetScan in blue and TarBase in green.
	Region	UTR3, CDS	Region of the mRNA where the interaction occurs.
	Binding Type	6mer; 7mer; 8mer; 9mer; miRNA bugle	The matching sites between the miRNA and the mRNA.
	Score	From 0 to 1	It is the site contribution score in the miTG score.
	Conservation	≥0	Number of species in which the predicted interaction is conserved.
	Signal-to-noise ratio (SNR)	>0	This score is a measure of the “signal to noise” ratio, which enables the identification of the miTG score of each interaction without background noise.
	Precision	From 0 to 1	This score is an indicator of the false-positive rate in a miTG interaction.
